# ACE2 and COVID-19 and the resulting ARDS

**DOI:** 10.1136/postgradmedj-2020-137935

**Published:** 2020-06-10

**Authors:** Xiaoqing Zhang, Shuren Li, Shaoqian Niu

**Affiliations:** Department of Cardiology, Hebei General Hospital, Shijiazhuang, China; Graduate School of Hebei Medical University, Shijiazhuang, China; Department of Cardiology, Hebei General Hospital, Shijiazhuang, China; Graduate School of Hebei Medical University, Shijiazhuang, China

## Abstract

This article reviews the correlation between ACE2 and COVID-19 and the resulting acute respiratory distress syndrome (ARDS). ACE2 is a crucial component of the renin-angiotensin system (RAS). The classical ACE-angiotensin Ⅱ (Ang II)-angiotensin type 1 receptor (AT1R) axis and the ACE2-Ang(1-7)-Mas counter-regulatory axis play an essential role in RAS system. ACE2 antagonises the activation of the classical RAS ACE-Ang II-AT1R axis and protects against lung injury. Similar to severe acute respiratory syndrome-related coronavirus, 2019 novel coronavirus (2019-nCoV) also uses ACE2 for cell entry. ARDS is a clinical high-mortality disease which is probably due to the excessive activation of RAS caused by 2019-nCoV infection, and ACE2 has a protective effect on ARDS caused by COVID-19. Because of these protective effects of ACE2 on ARDS, the development of drugs enhancing ACE2 activity may become one of the most promising approaches for the treatment of COVID-19 in the near future. In the meantime, however, the use of RAS blockers such as ACE inhibitors and angiotensin II receptor blockers that inhibit the damaging (ACE-Ang II) arm of the RAS cascade in the lung may also be promising. Trial registration number: NCT04287686.

In December 2019, a series of unexplained pneumonia appeared in Wuhan, China. Sequencing analysis of lower respiratory tract samples suggested that it was a new coronavirus and was named 2019 novel coronavirus (2019-nCoV) by WHO on 12 January 2020.^1^ On 11 February 2020, WHO officially named the disease caused by 2019-nCoV as COVID-19.^2^ The first 41 inpatients admitted to Wuhan Jinyintan Hospital from 16 December 2019 to 2 January 2020 were confirmed by the laboratory as 2019-nCoV infection, of which about 32% had diabetes, hypertension, cardiovascular disease and other underlying diseases. Common complications included acute respiratory distress syndrome (ARDS) (29%), RNAaemia (15%), acute heart injury (12%) and secondary infection (10%).^3^ Wang *et al*^4^ studied 138 patients with COVID-19 and found that common complications include ARDS (19.6%), shock (8.7%), arrhythmia (16.7%) and acute heart injury (7.2%). Guan *et al*^5^ included 1099 patients with COVID-19, of which 23.7% had at least one comorbidity. The most common symptoms were fever (43.8% on admission and 88.7% during hospitalisation) and cough (67.8%). On admission, ground-glass (56.4%) and patchy shadows (51.8%) were common imaging findings on chest CT . Lymphocytopenia was present in 83.2% of the patients on admission. During hospitalisation, 91.1% of patients received a diagnosis of pneumonia, followed by ARDS (3.4%) and shock (1.1%). In addition to common complications including ARDS in COVID-19, the latest research showed that the pathological findings of the patient’s lungs was consistent with ARDS. Xu *et al*^6^ completed the first pathological anatomy of a patient who died of COVID-19 and found that the patient presented with diffuse alveolar injury and hyaline membrane formation, which was consistent with the manifestation of ARDS. The pathological anatomic result was of great significance for the understanding and treatment of COVID-19.

The current outbreak of COVID-19 was similar to the severe acute respiratory syndrome (SARS) in Guangdong, China in 2002. Both of these events occurred during the winter, the first confirmed patient had exposure to wild animals before illness and both the outbreaks were caused by previously unknown coronavirus infection. COVID-19 has been confirmed to be caused by 2019-nCoV infection.^7^ The pathogen of SARS was SARS-related coronavirus (SARS-CoV). Coronavirus is named for its coronal protrusions composed of spike protein (S-protein) outside the viral lipid layer. Coronavirus binds to specific receptors on the surface of target cells through its S-protein, then enter host cells to replicate and cause infection.^8^ The S-protein of coronavirus contains two functional units, S1 and S2. S1 contains the receptor binding domain (RBD), which directly binds to ACE 2 (ACE2) that found to be the host receptor of SARS-CoV. S2 is responsible for fusion with the host cell membrane. When S1 binds to the host receptor ACE2, the cleavage site of S2 is exposed and cleaved by the host protease. This process is very important for virus infection.^9^ ACE2 is a type I membrane protein, which includes the N-terminal peptidase domain (PD) and the C-terminal collectrin-like domain (CLD). The PD of ACE2 provides a direct binding site for the coronavirus S-protein.^9^

## ACE2 and 2019-nCoV

ACE2 has been proved to be a key receptor for SARS-CoV S-protein binding.^10^ In the study regarding anti-SARS-CoV drugs, researchers have designed and discovered some small molecule compounds and peptides that can bind to the SARS-CoV- specific receptor ACE2, thus preventing SARS-CoV S-protein from binding to ACE2 and the fusion with the host cell membrane to avoid viral infection, suggesting that drugs can be designed aiming at the virus-acting receptor.^11^ Xu *et al*^7^ confirmed ACE2 as the receptor of 2019-nCoV by studying the binding capacity of the structural model of 2019-nCoV S-protein to human ACE2 receptor. However, the homology of S-protein between 2019-nCoV and SARS-CoV was relatively low. Although the amino acid sequence similarity between 2019-nCoV and SARS-CoV S-proteins was only 76.47%, the RBD domain of the two viral S-proteins was highly identical. Despite replacing four out of five important interface amino acid residues, 2019-nCoV S-protein was found to have a significant binding affinity to human ACE2. The 2019-nCoV and SARS-CoV S-protein shared an almost identical three-dimensional structure in the RBD domain, thus maintaining similar van der Waals and electrostatic properties in the interaction interface. This showed that the acting receptor of 2019-nCoV was the same as that of SARS-CoV. Zhou *et al*^12^ showed that ACE2 was essential for 2019-nCoV entering HeLa cells. The 2019-nCoV can infect HeLa cells expressing ACE2, but not HeLa cells that did not express ACE2. Xu *et al*^7^ and Zhou *et al*^12^ have shown that ACE2 was likely to be a mediating receptor for 2019-nCoV infecting host cells, so targeting ACE2 treatment may be effective for COVID-19.

Wrapp *et al*^13^ published the first electron micrograph of 2019-nCoV S-protein, which explained that 2019-nCoV makes use of highly glycosylated homotrimeric S-protein to enter host cells. The S-protein undergoes a variety of structural rearrangements to fuse with the host cell membrane, including S1 subunit binding to the host cell receptor, resulting in the instability of the S-protein trimer, the S1 subunit shedding and the S2 subunit forming a highly stable fusion structure. To access the host cell receptor, the RBD of the S1 subunit undergoes a hinge-like conformational movement to hide or expose key receptor binding sites. This study also found that the affinity of 2019-nCoV S-protein binding ACE2 is about 10–20 times higher than that of SARS-CoV S-protein, which was likely to explain why 2019-nCoV was highly infectious. In addition, this study tested three discovered SARS-CoV RBD-specific monoclonal antibodies S230, m396 and 80R and found that they do not have appreciable binding to 2019-nCoV S-protein, suggesting antibody cross-reactivity may be limited between the two RBDs. The above research results were of great significance for COVID-19 treatment.

Yan *et al*^9^ successfully presented the 2.9 Å resolution cryo-EM structure of full-length human ACE2 in complex with B0AT1, which is a neutral amino acid transporter whose surface expression in intestinal cells requires ACE2. This study found that ACE2 exists as a dimer and the PD of ACE2 have both open and closed conformations. Both conformations contain a mutual recognition interface with the coronavirus S-protein, so the dimeric ACE2 can simultaneously bind two trimeric S-proteins, which may be important for host cell membrane invagination and endocytosis of viral particles, this process is similar to endocytosis mediated by other receptors. Regarding the fusion of the viral S-protein with the host cell membrane, the C-terminal fragment of ACE2, especially the amino acid residues at positions 697–716, are cleaved by proteases such as transmembrane serine protease 2 (TMPRSS2), which can promote the viral S-protein to enter cells. The presence of B0AT1 may prevent TMPRSS2 from entering the cleavage site on ACE2. The distribution of ACE2 is wider than that of B0AT1. In addition to the kidney and intestine that mainly express B0AT1, ACE2 is also expressed in the lung and heart. Does this explain that the symptoms of viral infection mainly occur in the lungs without B0AT1 distribution? In addition, whether B0AT1 can indeed inhibit viral infection by blocking the cleavage of ACE2 remains to be further studied.

## The role of ACE2 in physiological and pathological pathway

### Discovery and distribution of ACE2

Donoghue *et al*^14^ and Tipnis *et al*^15^ identified ACE2 from the cDNA library of patients with heart failure and lymphoma in 2000, and since then opened a new branch of renin-angiotensin system (RAS). RAS is an important body fluid regulation system, which is mainly composed of two axes, namely ACE-angiotensin Ⅱ (Ang Ⅱ)-angiotensin type 1 receptor (AT1R) axis and ACE2-Ang(1-7)-Mas axis. ACE regulates blood pressure and the balance of water and sodium by binding to specific receptors AT1R and AT2R (AT1R plays a leading role). In addition, it has the effects of promoting inflammation, proliferation and vasoconstriction. ACE2 and ACE are structurally homologous, but their biological activities differ greatly. ACE2 mainly degrades Ang Ⅱ to produce Ang(1-7). The combination of Ang(1-7) and receptor Mas antagonises the ACE-Ang Ⅱ-AT1R axis, and plays a role in reducing blood pressure through vasodilation, resisting oxidative stress and cell proliferation.^16^

Previous studies have found that ACE2 is widely distributed in the heart, kidney, adipose tissue, vascular smooth muscle cells, brain tissue, testis, gastrointestinal tract, etc.^17^ Recently, Zhao *et al*^18^ exploited high-throughput single cell sequencing analysis technology to study a total of 43 134 lung cells, and found that ACE2 is expressed in 0.64% of lung cells, and most (about 83%) are concentrated in type Ⅱ alveolar cells (AT2), AT2 expressing ACE2 accounted for (1.4±0.4)% of the total and (1.4±0.4)% of ACE2+AT2 also express >30 functional genes related to virus assembly and replication. Therefore, 2019-nCoV seems to have cleverly evolved to take advantage of these AT2 cells for reproduction and spread. The wide expression of ACE2 in AT2 may explain the severe alveolar injury after infection. It is suggested that the prevention and treatment of COVID-19 can be started by inhibiting ACE2. In addition, ACE2 is also expressed in other cell types such as type I alveolar cells, bronchial epithelial cells, fibroblasts, endothelial cells and macrophages. The latest study by Zou *et al*^19^ constructed a 2019-nCoV infection risk map based on the expression level of ACE2 in various organs, and found that except lung, ACE2 is found in the respiratory tract, heart, kidney, oesophagus, ileum and bladder, which are at high risk of infection.

### ACE2 in the treatment and prognosis of ARDS caused by COVID-19

ARDS is a syndrome characterised by hypoxaemia and chest imaging suggests bilateral effusion but without evidence of heart failure. The risk factors for ARDS include pneumonia (bacteria, virus, fungus or opportunistic infection), inhalation of stomach contents and sepsis. The treatment is mainly based on protective mechanical ventilation and supportive treatment to avoid excessive fluid load. Nevertheless, the mortality rate of ARDS is still as high as 30%.^20^ Regarding ARDS caused by COVID-19, Liu *et al*^21^ included 109 patients with COVID-19, 48.6% of whom progressed to ARDS. Compared with patients without ARDS, patients with ARDS were older (aged 61 vs 49 years), more likely to suffer comorbidities (20.8% vs 1.8%) and the mortality rate was significantly higher (49.1% vs 8.9%). In addition, the clinical characteristics of patients with COVID-19 varied with the severity of ARDS, and the mortality rate of patients with moderate-to-severe ARDS was higher than that of mild ARDS. Antiviral, glucocorticoid or immunoglobulin treatment cannot significantly improve the survival rate of patients with ARDS caused by COVID-19.

In the progression of ARDS, ACE2 plays an important role. Relevant studies have shown that in the rat model of ARDS caused by lipopolysaccharide, the ACE activity of bronchoalveolar lavage fluid significantly increased, the corresponding expression of Ang Ⅱ increased and the expression of ACE2 and Ang(1-7) decreased.^22^ It is suggested that ARDS is due to RAS system imbalance, which was reflected in the increase of ACE-Ang II axis activity, and the decrease of ACE2-Ang(1-7) axis activity.

After the outbreak of SARS, many patients soon developed ARDS and died. Kuba *et al*^23^ found that the expression of ACE in SARS-CoV-infected mice did not change significantly, the expression of Ang Ⅱ increased significantly and the expression of ACE2 was downregulated. It is suggested that SARS-CoV interacts with ACE2 resulting in injury of lungs. Another study showed that in the mouse model of ARDS caused by bleomycin, the ACE2 gene-deficient mice had more severe symptoms than the control group, and after treatment with recombinant human ACE2 (rhACE2), the symptoms of lung injury significantly improved, and the survival rate also obviously improved. In addition, compared with mice with only ACE2 gene deficiency, the ARDS symptoms of applying angiotensin Ⅱ receptor blockers (ARB) or ACE2 gene-deficient mice with ACE gene knockout were milder. In addition, the lung injury in mice with AT1R deletion was less severe, while the lung injury in mice lacking AT2R was more severe. Therefore, during the occurrence of ARDS, the high expression of ACE, Ang II and AT1R aggravated the symptoms of lung injury, while ACE2 and AT2R improved the symptoms.^24^ Based on the mechanism of action of ACE2 in ARDS and the enlightenment of SARS, it can be considered that ACE2 has a protective effect on lung injury, and the downregulation of ACE2 is associated with more severe lung injury.

Due to the fact that SARS-CoV and 2019-nCoV both use ACE2 for cell entry, we can speculate that for COVID-19, ACE2 may also be downregulated by 2019-nCoV binding to ACE2, while the activation of the ACE-Ang II axis will promote lung injury. Imai *et al*^24^ found that rhACE2 protein treatment could reduce plasma Ang II levels and reduce lung injury in ACE2 knockout mice and wild-type mice, so we speculate that rhACE2 may become one of the most promising approaches for future treatment and improve the prognosis of patients with COVID-19. The goal of recombinant human ACE2 (GSK2586881) is to improve the concentration of ACE2 to rapidly improve arterial hypoxaemia and pulmonary circulatory haemodynamics of ARDS animal models. In human trials, GSK2586881 has been found to reduce Ang Ⅱ levels and increase Ang(1-7) levels; although failed to improve the physiological and clinical indicators of ARDS in patients, it did not promote adverse injury as a whole.^25^ This study proved that 44 patients with ARDS were well tolerated after using rhACE2, and they are most likely to represent the first clinical application in the field of ARDS.^26^ The potential of protecting lung injury by supplementing ACE2 has led to a clinical proof-of-concept study using rhACE2 in patients with COVID-19. It remains to be seen whether this approach supplementing the protective axis of the RAS will be efficacious against COVID-19.

### The potential therapeutic value of ACE inhibitor and ARB in COVID-19 and the resulting ARDS

**Figure 1:**
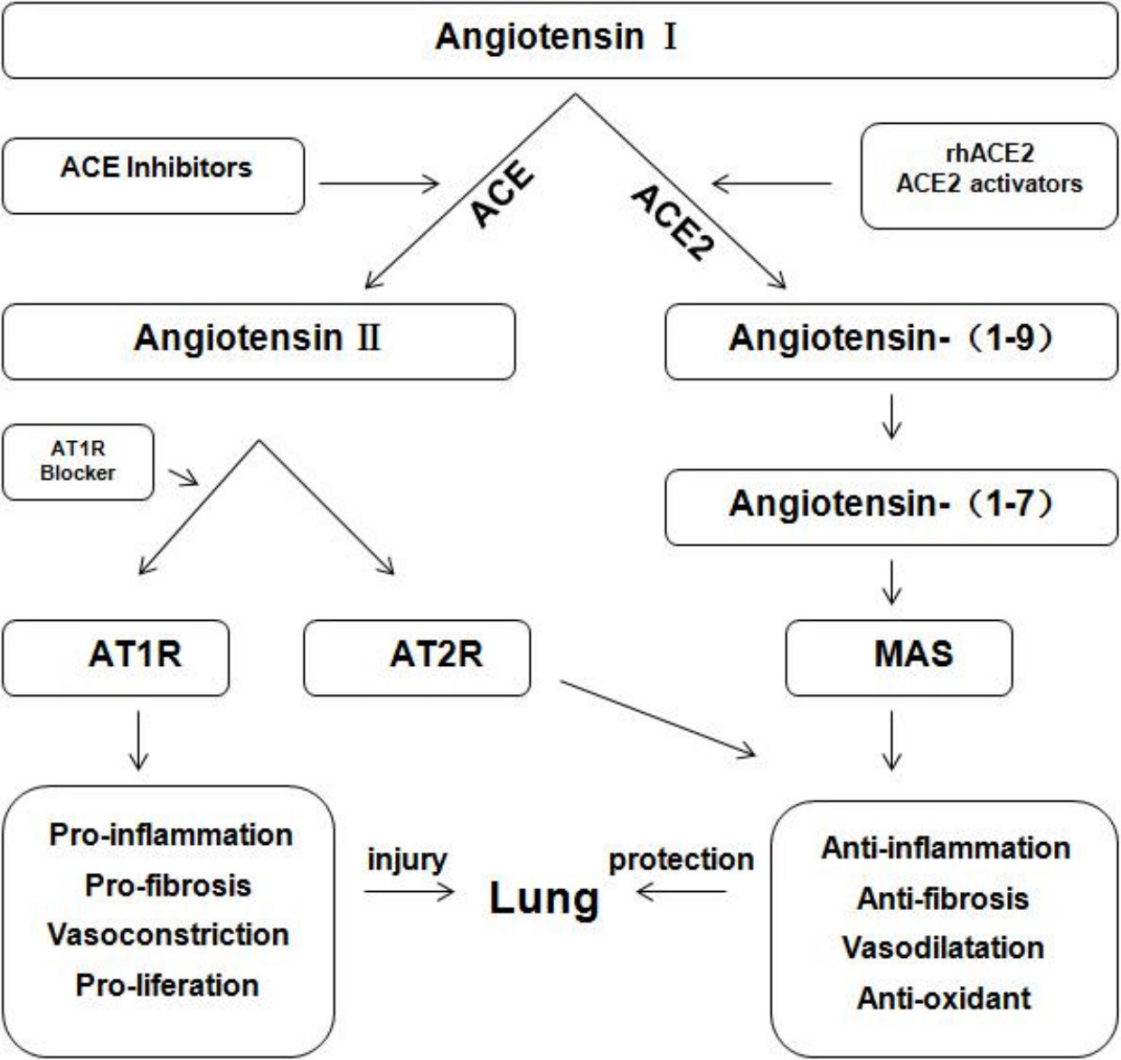
Schematic diagram of the renin-angiotensin system (RAS) in the lung showing the role of ACE2 as a key element in the counter-regulatory axis of the RAS. ACE2 opposes the harmful effects on lung injury of the ACE-angiotensin Ⅱ (Ang II)-angiotensin type 1 receptor (AT1R) axis by activating Mas and AT2R signalling. Pharmacological treatment with angiotensin Ⅱ receptor blockers (ARB) or ACE inhibitors (ACEI) will modulate several components of the RAS system. Treatment with ARB protects against lung injury by AT1R receptor blockade. rhACE2, recombinant human ACE2.

The timeline after the onset of COVID-19 was as follows: the median time from onset of symptoms to first hospital admission was 7 (4.0–8.0) days, to dyspnoea was 8 (5.0–13.0) days, to ARDS was 9 (8.0–14.0) days, to mechanical ventilation was 10.5 (7.0–14.0) days and to intensive care unit was 10.5 (8.0–17.0) days. The first 41 patients diagnosed in Wuhan all had pneumonia, and severe cases could progress to ARDS.^3^ In the study by Liu *et al*,^21^ 48.6% of patients with COVID-19 progressed to ARDS. Although there is currently no effective drug for the treatment of COVID-19 and the resulting ARDS, however, rhACE2 is currently considered as a therapeutic approach to act as a decoy halting the interaction between 2019-nCoV and ACE2 to lessen viral entry. In the meantime, however, the use of RAS blockers such as ACE inhibitors (ACEI and ARB that inhibit the damaging (ACE-Ang II) arm of the RAS cascade in the lung may also be promising. Indeed, relevant studies have confirmed the protective effect of ACEI or ARB on lung injury. Liu *et al*^27^ found that captopril can reduce pulmonary hypertension, delay the progression of ARDS and protect lung vascular endothelial cells by applying captopril in a rat model of oleic acid-induced ARDS. Neyrinck *et al*^28^ also confirmed the therapeutic effect of ACEI on endotoxin-induced lung injury in rats. Mortensen *et al*^29^ showed that continuous administration of ACEI in hospital can reduce the mortality and intubation rate of patients with common viral pneumonia. Wösten *et al*^22^ showed that the application of ARB also had a therapeutic effect for ARDS. The above research suggests that in the absence of specific therapeutic drugs against 2019-nCoV infection, ACEI or ARB may be considered to reduce lung pathological damage in infected patients, but the specific efficacy needs to be further verified in future clinical trials. In addition, there is currently a different opinion that RAS inhibitors will reflexively increase the expression of ACE2, which theoretically has the effect of accelerating viral replication or entering cells, and ACEI/ARB should be discontinued. However, there is no research to confirm that the increased expression of ACE2 can increase the chance of viral infection, so there is no conclusive evidence to confirm that patients with COVID-19 with cardiovascular disease should discontinue RAS inhibitors.^30^ Kreutz *et al* stated that a critical review of available evidence does not support a deleterious effect of RAS inhibitors in COVID-19. Therefore, there is currently no reason to discontinue RAS inhibitors in stable patients facing the COVID-19 pandemic.^31^ The American College of Cardiology also declared a statement that COVID-19 with hypertension, heart failure and ischaemic heart disease should not discontinue ACEI/ARB.^32^ Mancia *et al* provided evidence that ACEI/ARB did not modify the susceptibility to COVID-19, and neither ACEI nor ARB showed an independent association with COVID-19 in patients with mild-to-moderate disease or in those with severe disease.^33^ Mehra *et al* found some independent factors associated with an increased risk of in-hospital mortality for COVID-19 were an age >65 years, coronary artery disease, heart failure, cardiac arrhythmia, chronic obstructive pulmonary disease and current smoking. No increased risk of in-hospital mortality was found to be associated with the use of ACEI or the use of ARB in COVID-19.^34^ Thus, there was neither an independent relationship between ACEI/ARB and the susceptibility to COVID-19, nor between RAS inhibitors among the clinical progress of COVID-19. Thus, ACEI/ARB appears reasonable, rather than harmful to COVID-19 (figure 1).

## Summary

At present, the first pathological anatomical result of COVID-19 death patients provided preliminary evidence for lung injury in patients with 2019-nCoV infection, but the specific mechanism still needs to be further studied. Since 2019-nCoV and SARS-Cov have a high degree of homology, it is speculated that the pathogenesis of lung injury caused by the two viruses is similar. A deeper understanding of the pathogenesis is conducive to timely and effective intervention in epidemic prevention and control. From the downregulation of ACE2 following SARS-CoV binding to ACE2 and resulting in excessive activation of RAS and exacerbated pneumonia progression, we speculate that rhACE2 which can increase ACE2 levels may become one of the most promising approaches for future treatment and improve the prognosis of patients with COVID-19. In the meantime, however, the use of RAS inhibitors such as ACEI and ARB that inhibit the damaging (ACE-Ang II) arm of the RAS cascade in the lung may also be promising. By now, no relevant studies have strongly confirmed that 2019-nCoV infection will cause downregulation of ACE2, so more clinical studies are needed to confirm the specific mechanism between 2019-nCoV-ACE2-ARDS.

**Key references**

- Chen LN, Yang XH. Homeostasis of the local renin-angiotensin system and acute lung injury. *Progr Physiol Sci* 2013;44:133–7.
- Wösten-van Asperen RM, Lutter R, Specht PA, *et al*. Acute respiratory distress syndrome leads to reduced ratio of ACE/ACE2 activities and is prevented by angiotensin-(1-7) or an angiotensin II receptor antagonist. *J Pathol* 2011;225:618–27.
- Kuba K, Imai Y, Rao S, *et al*. A crucial role of angiotensin converting enzyme 2 (ACE2) in SARS coronavirus-induced lung injury. *Nat Med* 2005;11:875–9.
- American College of Cardiology. HFSA/ACC/AHA statement addresses concerns re:using RAAS antagonists in COVID-19. [EB/OL]. ACC News Story. Available: https://viajwat.ch/2REZU2H.
- Mancia G, Rea F, Ludergnani M, *et al*. Renin-Angiotensin-Aldosterone system blockers and the risk of Covid-19. *N Engl J Med* 2020.

**Self assessment questions**

- What is the similarity and difference between 2019-nCoV and SARS-CoV?
  - Both use ACE2 for cell entry.
  - The amino acid sequence similarity between 2019-nCoV and SARS-CoV S-proteins was 87%.
  - The affinity of 2019-nCoV S-protein binding ACE2 is about 10–20 times higher than that of SARS-CoV S-protein.
  - SARS-CoV RBD-specific monoclonal antibodies do not have appreciable binding to 2019-nCoV S-protein.
- Where are ACE2 receptors located in the body?
  - Lungs.
  - Heart.
  - Kidney.
  - Oesophagus, ileum.
- Regarding the RAS system, which is correct?
  - RAS system is mainly composed of two axes, namely ACE-Ang Ⅱ-ATIR axis and ACE2-Ang(1-7) -Mas axis.
  - ACE2 and ACE are structurally and biologically homologous.
  - The ACE-Ang Ⅱ-AT1R axis plays a role in reducing blood pressure through vasodilation, resisting oxidative stress and cell proliferation.
  - The high expression of ACE, Ang II and AT1R aggravated the symptoms of lung injury, while ACE2 and AT2R improved the symptoms.
- Regarding the application of ACEI or ARB in COVID-19, which is correct?
  - The application of ACEI/ARB increases the susceptibility to COVID-19.
  - The use of ACEI/ARB showed an independent association with COVID-19 in patients with mild-to-moderate disease or in those with severe disease.
  - No increased risk of in-hospital mortality was found to be associated with the use of ACEI or the use of ARB in COVID-19.
  - There is no research to confirm that the increased expression of ACE2 can increase the chance of viral infection.
- Regarding the application of recombinant human ACE2 (rhACE2) in ARDS, which is correct?
  - rhACE2 protein treatment could reduce plasma Ang II levels and reduce acute lung injury of animals model.
  - rhACE2 may become one of the most promising approaches for future treatment and improve the prognosis of patients with COVID-1
  - GSK2586881 improved the physiological and clinical indicators of ARDS in patients.
  - It remains to be seen whether this approach supplementing the protective axis of the RAS will be efficacious against COVID-19 and the resulting ARDS.

**Answers**

- (A), (C), (D)
- (A), (B), (C), (D)
- (A), (D)
- (C), (D)
- (A), (B), (D)
